# Physical Examination as a Clue to Transthyretin Cardiac Amyloidosis: A Case Report

**DOI:** 10.7759/cureus.42925

**Published:** 2023-08-03

**Authors:** Tatsuhiro Hirano, Mayumi Takeoka, Michiyo Yamano, Tatsuya Kawasaki

**Affiliations:** 1 Cardiology, Matsushita Memorial Hospital, Moriguchi, JPN; 2 Central Clinical Laboratory, Matsushita Memorial Hospital, Moriguchi, JPN

**Keywords:** attr amyloidosis, attr, attr cardiac amyloidosis, transthyretin cardiac amyloidosis, popeye's sign, physical examination, fourth sound, diagnosis, amyloidosis

## Abstract

Transthyretin (ATTR) cardiac amyloidosis has recently received increased attention; however, the diagnosis is often delayed. We present a case of ATTR cardiac amyloidosis in which a comprehensive history-taking and focused physical examination played an important role in establishing the diagnosis. A 75-year-old man was referred to the cardiology department for left ventricular hypertrophy on electrocardiography. No fourth sound was audible despite concentric biventricular hypertrophy and diastolic dysfunction on echocardiography. Additional history-taking revealed that he had undergone bilateral carpal tunnel syndrome surgery almost 35 years earlier and had a biceps tendon rupture about 15 years earlier; bunching of the arm on flexion, or Popeye's sign, was noted. Technetium-99m-pyrophosphate showed diffuse uptake not only in both ventricles but also in both atria. The findings were consistent with the absence of the fourth sound. The present case highlights the importance of a focused physical examination as well as history-taking as a clue to ATTR cardiac amyloidosis in patients with unexplained left ventricular hypertrophy.

## Introduction

Transthyretin (ATTR) cardiac amyloidosis has more recently gained increasing attention because of the establishment of a non-invasive diagnostic technique and fundamental treatment [1,2]. However, the diagnosis is often delayed since this condition develops at an older age with various confounding comorbidities [3, 4]. We report a case of ATTR cardiac amyloidosis in which focused history-taking and physical examination played a pivotal role in the diagnosis.

## Case presentation

An asymptomatic 75-year-old man was referred to the cardiology department for left ventricular hypertrophy on electrocardiography. His medical history was notable for transurethral resection for bladder cancer, catheter ablation for atrial fibrillation, well-controlled hypertension, and hyperuricemia. His medications included rivaroxaban 15 mg daily, amlodipine 5 mg daily, and febuxostat 20 mg daily. He drank three cans of beer daily, had quit smoking two years earlier with a 20-pack-year smoking history, did not use illicit drugs, and had no known allergies. There was no family history of heart disease.

**Figure 1:**
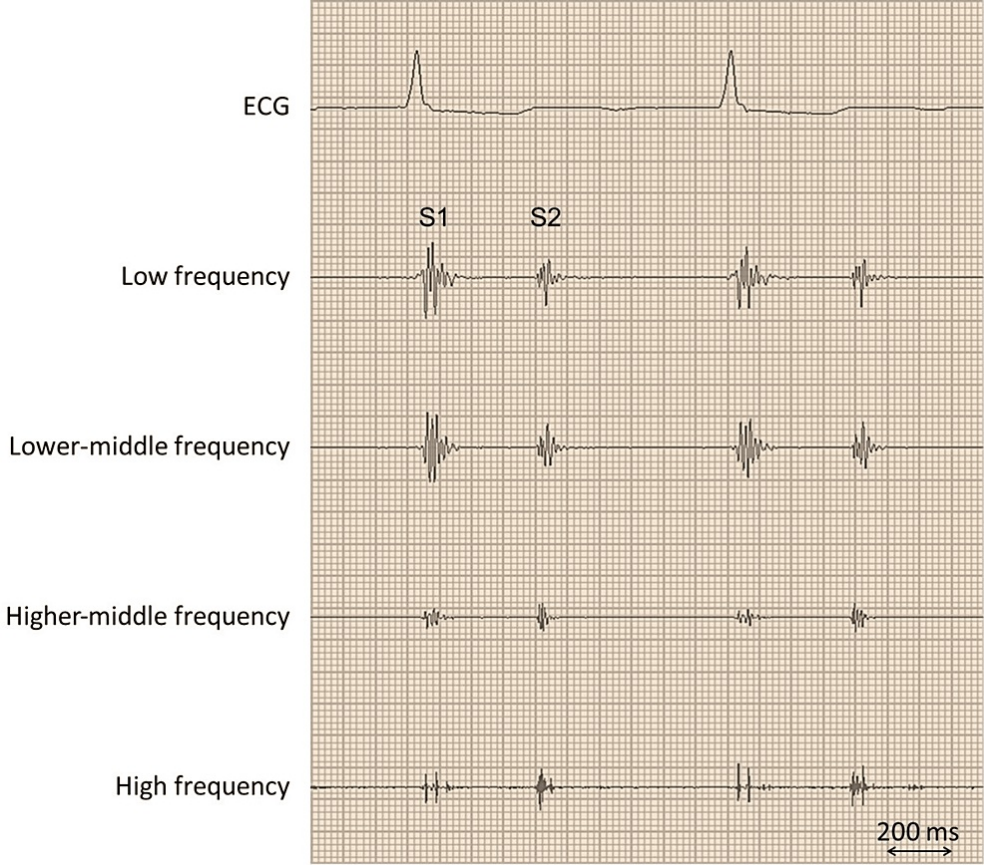
The patient's phonocardiography report No additional sound, including the fourth sound, is noted on a phonocardiogram recorded on the apex. S1 denotes the first sound; S2 is the second sound.

On examination, his blood pressure was 122/96 mmHg, his heart rate was 79 beats per minute, his respiratory rate was 18 breaths per minute, and his oxygen saturation was 98% while he was breathing ambient air. The jugular venous pressure was not elevated, and neither a heart murmur nor an additional heart sound, including the fourth heart sound, was audible (Figure 1).

Both lungs were clear on auscultation, and there was no edema in his legs.

Electrocardiography showed left ventricular hypertrophy with an R height in lead V_5_ of 3.07 mV and poor R-wave progression in the precordial leads. Chest radiography was normal. The complete blood cell count was normal, as were the electrolyte balance, creatinine kinase level, thyroid functions, albumin level, and renal and liver function tests. The high-sensitivity cardiac troponin T level was 0.030 ng/mL (reference value, ≤0.100), and the brain natriuretic peptide level was 204 pg/mL (reference value, ≤18.4).

Echocardiography showed a left ventricular ejection fraction of 55% with diffuse hypertrophy in both the left and right ventricles (i.e., approximately 15 mm and 5 mm, respectively). The left ventricular mass index was 111.9 g/ms^2^ (reference value: 56 to 92 ms^2^). Tissue Doppler imaging showed the ratio of the mitral valve E to the A wave of 2.66 and the deceleration time of the E wave of 209 ms. The ratio of mitral E to early diastolic mitral annular tissue velocity (E/e') was 32.0. There were no findings suggestive of significant valvular heart disease, ventricular outflow tract obstruction, or shunt disease. A diagnosis of diastolic dysfunction along with concentric hypertrophy was made. Two-dimensional speckle-tracking echocardiography showed a global longitudinal strain of -12.4% with an apical sparing pattern.

**Figure 2:**
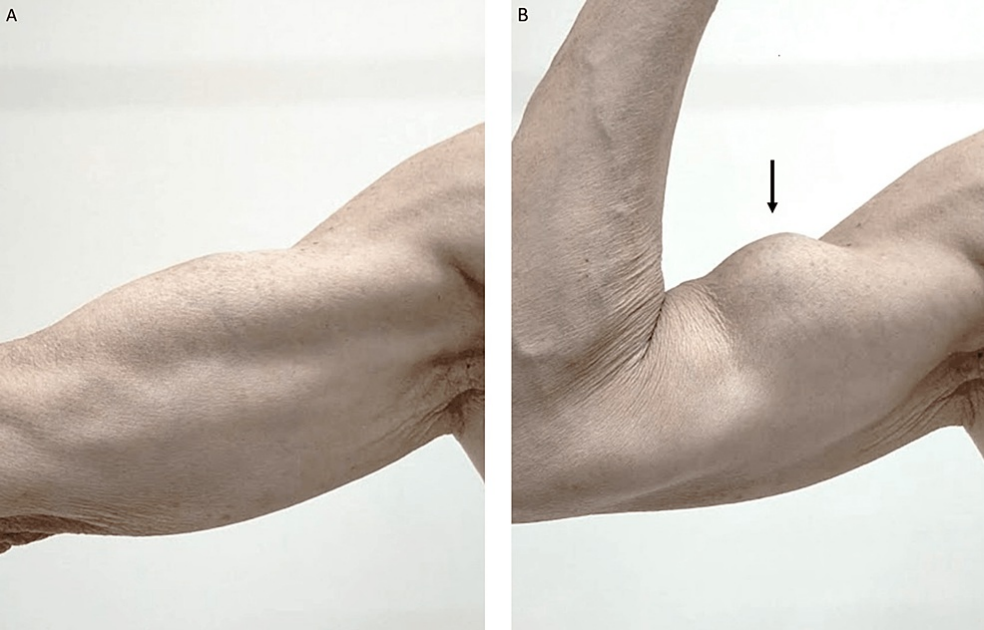
A photograph of the patient's right upper arm In comparison with the extension of his arm (A), a bulge is noted on the right upper arm in flexion (B, arrow), a finding known as Popeye's sign, which is a hallmark of biceps tendon rupture.

Additional history-taking showed that the patients had received bilateral surgery for carpal tunnel syndrome almost 35 years earlier. In addition, approximately 15 years earlier, a biceps tendon rupture developed while attempting to carry a 20-kg barrel. A bunching of the biceps when the patient flexed his arm, known as Popeye's sign, was noted (Figure 2).

**Figure 3:**
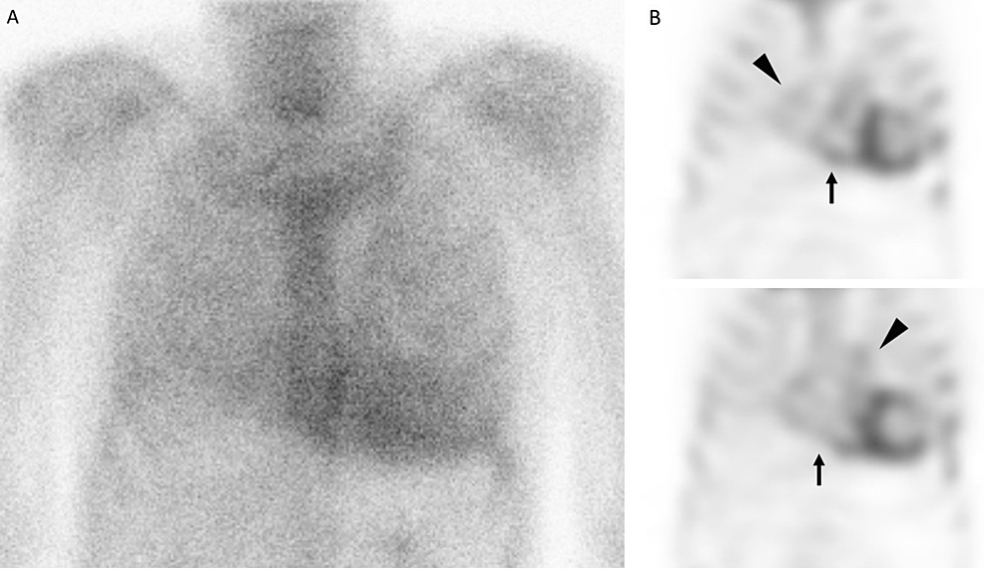
The patient's scintigraphy images Myocardial imaging with technetium-99m-pyrophosphate demonstrates more tracer uptake of the heart than the bones (A). Coronal images show tracer uptake not only in the left ventricle but also in the right ventricle (B, arrows), along with biatrial uptake (B, arrowheads).

Technetium-99m-pyrophosphate showed diffuse uptake not only in both ventricles but also in both atria (Figure 3), which was more severe than those in the bones, and no monoclonal proteins were detected, findings consistent with ATTR cardiac amyloidosis [5].

Neither a tissue biopsy nor genetic analysis was performed for his preference. The patient has been doing well for more than a year, although he underwent a second session of catheter ablation for recurrent atrial fibrillation.

## Discussion

The patient was referred to the cardiology department for left ventricular hypertrophy on electrocardiography and was found to have surgery for carpal tunnel syndrome. Furthermore, physical examination, including Popeye’s sign and lack of fourth heart sound despite a stiff ventricle, was useful in suspecting ATTR cardiac amyloidosis. Although pathological or genetic analyses were not performed, it was reasonable to make a diagnosis of ATTR cardiac amyloidosis in this patient given the excellent diagnostic value of technetium-99m-pyrophosphate scintigraphy [5].

In this case, the diagnosis of ATTR cardiac amyloidosis was made approximately 35 years after carpal tunnel syndrome. Patients with ATTR cardiac amyloidosis often experience a delay in diagnosis, with a median duration of approximately six years [3], which can lead to adverse outcomes. In particular, a diagnosis of wild-type ATTR cardiac amyloidosis is challenging since the condition develops at an older age with various confounding comorbidities in the absence of supportive blood biomarkers [3, 4], such as angiotensin-converting enzyme for sarcoidosis or alpha-galactosidase for Fabry disease. Although a non-invasive diagnosis of ATTR cardiac amyloidosis using bone scintigraphy has been strongly proposed [5], the absence of specific echocardiographic features routinely obtained [6] is unlikely to lead physicians to choose an expensive diagnostic test like scintigraphy that is available in limited facilities. Therefore, a focused history and physical examination are important for early diagnosis in patients with significant left ventricular hypertrophy.

Popeye's sign, a large bulge on the upper arm when flexed, was observed in this patient. This sign, named after the fictional cartoon character's spherical biceps, is well known in orthopedics as a hallmark of biceps tendon rupture. More recently, Popeye's sign has gained attention in other medical fields, including cardiology, because non-traumatic biceps tendon ruptures sometimes develop in patients with ATTR cardiac amyloidosis. A prospective registry of patients with cardiac amyloidosis showed that Popeye's sign was observed in 35.7% of 143 patients, including 128 with ATTR cardiac amyloidosis [7]. In another cohort of 111 patients with ATTR cardiac amyloidosis, spontaneous rupture of the distal biceps tendon was observed in 33.3% of patients (dominant arm in 95% and bilateral in 24.3%), which developed a median of five years before the diagnosis of heart failure [8]. In our case, the diagnosis of ATTR cardiac amyloidosis was made approximately 15 years after a non-traumatic rupture of the biceps tendon. The pathogenesis linking amyloidosis and Popeye's sign remains to be fully elucidated, with possible mechanisms including ATTR deposition in tendons and ligaments [9].

The patient did not have a fourth heart sound in the setting of sinus rhythm, despite a "stiff ventricle" suggested by the significant left ventricular hypertrophy and diastolic dysfunction on echocardiography. It is worth noting that, in patients with amyloidosis, the fourth heart sound is rare due to diminished atrial kick as a result of amyloid infiltration in the atria [10,11], whereas a majority of sinus patients with hypertrophic cardiomyopathy show the fourth heart sound (e.g., 75% [12]). The fourth heart sound can be difficult to detect by auscultation [13], but in our patient, phonocardiography was used to assess the presence or absence of additional heart sounds. In the present patient, the atrial function was considered to be impaired, given the low mitral A-wave on echocardiography and previous history of atrial fibrillation. In addition, scintigraphic images suggested ATTR infiltration not only in the ventricles but also in the bilateral atria.

## Conclusions

The present case highlights the importance of not only history-taking (e.g., carpal tunnel syndrome) but also a focused physical examination (e.g., Popeye’s sign and absence of the fourth heart sound despite the stiff ventricle) as a clue to ATTR cardiac amyloidosis in patients with unexplained left ventricular hypertrophy.
